# Day2day: investigating daily variability of magnetic resonance imaging measures over half a year

**DOI:** 10.1186/s12868-017-0383-y

**Published:** 2017-08-24

**Authors:** Elisa Filevich, Nina Lisofsky, Maxi Becker, Oisin Butler, Martyna Lochstet, Johan Martensson, Elisabeth Wenger, Ulman Lindenberger, Simone Kühn

**Affiliations:** 1 Center for Lifespan Psychology, Max Planck Institute for Human Development, Lentzeallee 94, 14195 Berlin, Germany; 2 Clinic and Policlinic for Psychiatry and Psychotherapy, University Clinic Hamburg-Eppendorf, Martinistraße 52, 20246 Hamburg, Germany; 3 Department of Psychology, Lund University, Box 117, 221 00 Lund, Sweden

**Keywords:** MRI, Variability, Reliability, Structural imaging, Resting state, Ergodicity, Longitudinal design

## Abstract

**Background:**

Most studies of brain structure and function, and their relationships to cognitive ability, have relied on *inter*-individual variability in magnetic resonance (MR) images. *Intra*-individual variability is often ignored or implicitly assumed to be equivalent to the former. Testing this assumption empirically by collecting enough data on single individuals is cumbersome and costly. We collected a dataset of multiple MR sequences and behavioural covariates to quantify and characterize intra-individual variability in MR images for multiple individuals.

**Methods and design:**

Eight participants volunteered to undergo brain scanning 40–50 times over the course of 6 months. Six participants completed the full set of sessions. T1-weighted, T2*-weighted during rest, T2-weighted high-resolution hippocampus, diffusion-tensor imaging (DTI), and proton magnetic resonance spectroscopy sequences were collected, along with a rich set of stable and time-varying physical, behavioural and physiological variables. Participants did not change their lifestyle or participated in any training programs during the period of data collection.

**Conclusion:**

This imaging dataset provides a large number of MRI scans in different modalities for six participants. It enables the analysis of the time course and correlates of intra-individual variability in structural, chemical, and functional aspects of the human brain.

## Background

Magnetic resonance (MR) imaging can non-invasively quantify different aspects of brain structure with high spatial resolution. Different structural MR sequences are optimized to distinguish between different tissue types. For example, T1 and T2-weighted images distinguish between white and grey matter and cerebrospinal fluid (CSF). T2*-weighted images distinguish between different oxygen levels in brain tissue and are therefore an indirect measure of brain function [1]. Diffusion-tensor images (DTI) quantify water diffusivity and can therefore map major white matter tracts [2]; and proton magnetic resonance spectroscopy can be optimally set to measure the concentration of chemical groups, typically contained in neurotransmitters [3].

Within the last decades, neuroscientists have investigated different aspects of brain organization. First, associations between behaviour—e.g. cognitive ability, personality characteristics—and measures of grey matter thickness or density, white matter integrity, and functional connectivity between different brain areas have been identified in primarily cross-sectional studies [4]. The inter-individual variability in brain structure and function present in the normal population can explain some portion of variability in different cognitive processes, from low-level visual processing [5] to high-level cognition [6]. Further, clinical studies have identified large differences in these neurophysiological measures between patients and healthy control individuals [7]. Taken together, these results tend to paint a predominantly static, or trait-like, picture of brain organization and its association with behavior.

At the same time, other research, mostly based on the same measures, has revealed that the human brain is plastic [8–10]. For instance neuroimaging studies in clinical populations revealed that the course of disease, as in schizophrenia, is related to the trajectory of individual neural change [11]. Intervention studies have shown changes in brain structure and function in healthy individuals following diverse forms of explicit and often intense training [8, 12]. More strikingly, measures of brain structure and function have also been shown to depend on daily physiological variations that occur even in non-experimental settings like exercise [13], water [14, 15] and caffeine intake [16–18] or the menstrual cycle [19], revealing potentially important sources of *intra*-*individual* variability.

**Fig. 1:**
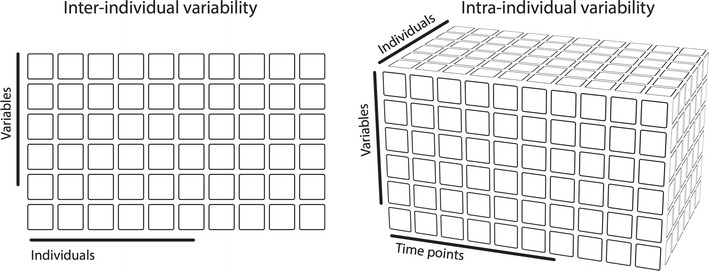
Main differences between the Day2day dataset and other existing datasets. Most available MRI datasets that we are aware of investigate inter-individual variability only. By collecting MR data from the same participants across multiple occasions, we provide a dataset to investigate intra-individual variability (see also [23])

Cattell [20] usefully framed inter- and intra-individual variability with the concept of the Data Box, in which each data point represents the person under scrutiny, the specific measure taken, and the occasion (time point) at which the measure was taken [21, 22] (see Fig. 1). Importantly, in so-called ergodic data sets, that is, data sets that are drawn from a homogeneous population and are stationary, slicing this data box along different dimensions will lead to equal results [23]. But the variation of brain measures across individuals and time suggests that MR data might violate both assumptions. When data are non-ergodic, the structure of within-person variations over time cannot be derived from the structure of between-person differences at any given point in time. Hence, imaging results expressing between-person differences cannot be generalized to apply to within-person time series [23, 24].

To summarize, cross-sectional data sets focus on between-person differences. Similarly, longitudinal data sets with few occasions, such as pretest–posttest intervention studies, focus on between-person differences in within-person change. Both research designs assume that individuals are drawn from a homogeneous population, and that the data structure does not change over time. Whether these assumptions are justified can only be known when between-person differences are compared to within-person changes. Hence, *intra*-individual variability must be studied explicitly, and appropriate methods for this purpose must be developed. Here, we report a dataset in which the same participants underwent MRI scanning on multiple (40–50) occasions. To the best of our knowledge, only one previous study collected imaging data of the same (single) individual with a large number of repetitions (MyConnectome project [18, 25]). The dataset presented here consists of a large number of scans for *multiple* individuals and is made available for public scientific use. It includes additional sequences and further behavioural covariates that make it comparable and complementary to the MyConnectome project and others of this nature.

## Construction and content

### Participants

Eight participants (2 male, mean age 29 years, SD = 2.58, range 24–32) volunteered to contribute to the dataset, for which they were scanned 40–50 times over the course of 6–8 months. Two of the participants (one male, one female) did not find the time to complete data acquisition. All participants were free of psychiatric disorder and had never previously suffered from a mental disease (see Table 1 for further details).

**Table 1:** Summary of scanning sessions

ID	Days to complete	Total sessions	Avg. interval (days)	T1/RS	Hipp	DTI	SVS
1	168	50	3.4	50	50	25	9
2	107	13	8.2	13	13	6	0
3	394	50	7.9	50	49	41	15
4	56	11	5.1	11	10	8	0
5	208	46	4.5	45	45	17	14
6	170	47	3.6	47	46	22	13
7	218	43	5.1	43	43	29	11
8	232	50	4.6	49	49	24	10

Days to complete indicates the total days spanned between the first and last scanning sessions. The last four columns indicate the total number of images acquired for each sequence

*T1/RS* T1-MPRAGE sequences/resting state (EPI), *Hipp* high-resolution hippocampus, *DTI* diffusion-tensor imaging, *SVS* single-voxel spectroscopy

### Scanning procedure

Images were collected on a 3T Magnetom Trio MRI scanner system (Siemens Medical Systems, Erlangen, Germany) using a 12-channel radiofrequency head coil. Participants kept their eyes closed during all image acquisitions.

#### T1 sequence

Structural images were collected using a three-dimensional T1-weighted magnetization prepared gradient-echo sequence (MPRAGE) with the following parameters: TR = 2500 ms, TE = 4.77 ms, TI = 1100 ms, FOV = 256 × 256 × 192 mm^3^, flip angle = 7°, bandwidth = 140 Hz/pixel, 1 × 1 × 1 mm^3^ voxel size, 9:20 min duration.

#### Resting state sequence

Functional images were collected using a T2*-weighted echo planar imaging (EPI) sequence sensitive to blood oxygen level dependent (BOLD) contrast with the following parameters: TR = 2000 ms, TE = 30 ms, FOV = 216 × 216 × 129 mm^3^, flip angle = 80°, bandwidth = 2042 Hz/pixel, voxel size 3 × 3 × 3 mm^3^, distance factor = 20%, 36 axial slices using GRAPPA acceleration factor 2, 5:08 min duration.

#### Hippocampus sequence

A high resolution T2-weighted turbo spin echo sequence was used to acquire high-resolution images of the hippocampus, to estimate subfield volumes with the following parameters: TR = 8150 ms, TE = 50 ms, 0.4 × 0.4 mm in plane resolution, 2 mm slice thickness, FOV = 150 × 150 × 62 mm^3^, flip angle = 120°, bandwidth = 99 Hz/pixel, turbo factor = 15, 31 slices covering the anterior three quarters and in some cases the whole hippocampus [26], 7:13 min duration.

#### Diffusion tensor imaging sequence

Diffusion-weighted images were obtained with a single-shot diffusion-weighted spin-echo-refocused echo-planar imaging sequence with the following parameters: TR = 8000 ms, TE = 93 ms, FOV 224 × 224 × 124 mm^3^, voxel size 2 × 2 × 2 mm^3^, 62 slices using GRAPPA acceleration factor 2, b-value 1000 s/mm^2^, 60 directions, 9:22 min duration.

#### Magnetic resonance spectroscopy sequence

To measure Proton (1H) magnetic resonance spectroscopy we used a point resolved spectroscopy (PRESS) sequence with the following parameters: TR = 3000 ms, TE = 80 ms, 128 averages, 90° flip angle, automatic shimming (advanced), vector size = 2048, 128/8 spectra with and without water-suppression, respectively, 6:36 min duration. All MRS voxels were individually positioned by anatomically trained MR operators on the bilateral ACC using the high-resolution three-dimensional T1-weighted MPRAGE (see above), which was collected in the same session.

### Scanning schedule

**Fig. 2:**
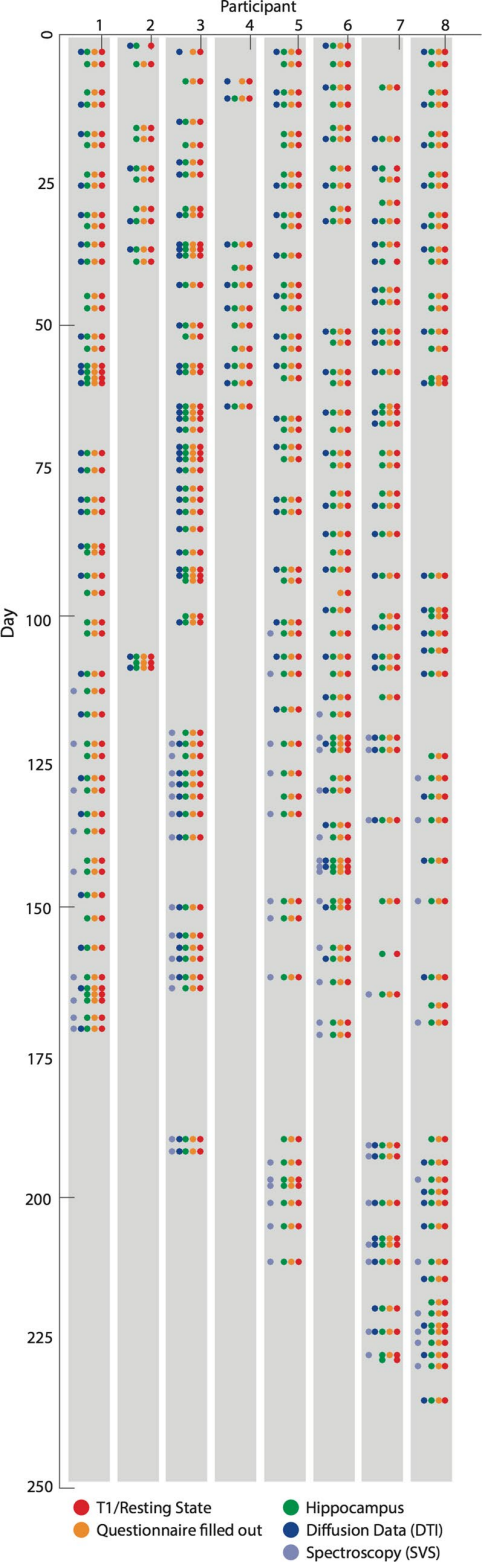
Overview of scans for each participant and MR quality control through phantom scanning. *Each row* represents one participant, and *each vertical line* corresponds to one scanning instance. *Colours* (see legend) represent the MR sequences collected

Data collection took place between July 2013 and February 2014. While we aimed at collecting MR images from each participant two to three times a week to capture short-term variability, each participant was free to arrange a scanning regime that would optimally fit with his or her schedule. Additionally, scanning depended on availability of the MR scanner. As a result, MR data collection was not always done at regular intervals. Figure 2 provides an overview of the timing of each scanning session for each participant.

### Covariates collected

We collected a number of covariates (see Table 2) related to scanner status, behaviour, and affect of the participants during the scan and in the 24 h prior to scanning. We aimed at collecting information for those covariates that had been shown or were expected to affect measures of brain structure and function. Participants did not modify their habits or engaged in any intensive training programs during the period of data collection, so the range of daily variation in covariates can be expected to roughly match that of the normal population.

**Table 2:** List of covariates collected for each of the sessions

Covariate	Variable name	Comment
General		
Date	*scanDate*	Expressed in format ‘dd. mm. yyyy’
Time	*scanTime*	Time of start of scanning session
Minimum outside temperature for the day of the scan (°C)	*minTemp_C*	All weather variables were collected from the German Weather Service [27]
Maximum outside for the day of the scan temperature (°C)	*maxTemp_C*	
Wind (km/h)	*wind_km_h*	
Precipitation (mm)	*precip_mm*	
Kind of precipitation	*precip_form*	Precipitation codes are as follows: 0: No precipitation 1: Only scattered precipitation 2: Only scattered precipitation in liquid form 3: Only scattered precipitation in frozen form 6: Rain or drizzle 7: Precipitation in frozen form 8: Precipitation in liquid and frozen form 9: Measurement error
Atmospheric pressure (hp)	*atmPressume*	
Relative humidity (%) (daily average)	*relHumidity*	
Hours of sunshine	*sunshine_hrs*	
Scanner characteristics		
MR room temperature (°C)	*MR_Room_Temperature*	
MR room humidity (%)	*MR_Room_Humidity*	
MR helium level (%)	*MR_HeliumLevel*	
Behaviour during scan		
Slept during scan	*sleptDuringScan*	Binary variable (0/1)
Rumination	*ruminationDuringScan*	1–6 Likert scale
Anxiety	*anxietyDuringScan*	1–6 Likert scale
Physiological variables		
Day of menstrual cycle	*menstrualCycleDay*	Since first day of last period. Additional information is available on request
Caffeine intake in the last 24 h	*caffein_last24hs_cups*	In number of cups of coffee
Caffeine intake in the last 2 h	*caffein_last2hs_cups*	In equivalent number of cups of coffee
Chocolate intake (g) in the last 24 h	*chocolate_last24hs_gramms*	
Cacao intake (%) in the last 24 h	*chocolate_last2hs_gramms*	
Chocolate intake (g) in the last 2 h	*cacao_last24hs_percentage*	
Cacao intake (%) in the last 2 h	*cacao_last2hs_percentage*	
Alcohol intake in the last 24 h	*alcohol_last24hs_drinks*	In number of alcoholic drinks
Cigarettes smoked in the last 24 h	*cigarettes_last24hs*	
Marihuana consumed in the last 24 h (in number of cigarettes)	*marihuanaCigarettes_last24hs*	
Liquid intake in the last 24 h	*liquid_last24hs_liters*	
Sweets intake in the last 24 h	*sweets_last24hs*	1–6 Likert scale
Salt intake in the last 24 h	*salt_last24hs*	1–6 Likert scale
Weight (kg)	*weight_kg*	Weight was measured without shoes but otherwise with clothes on
Blood pressure (mmHg, systolic and diastolic)	*bloodPressure_systolic_mmHg, bloodPressure_diastolic_mmHg*	
Saliva estrogen		
Saliva testosterone		
Physical pain	*physicalPainDuringScan*	Experienced during scan, 1–6 Likert scale
General health rating	*generalHealth_last24hs*	Subjective rating of the last 24 h, 1–6 Likert scale
General stress rating	*generalStress_last24hs*	Subjective rating of the last 24 h, 1–6 Likert scale
Behavioural and affective variables		
Ease of concentration	*easeOfConcentration_last24hs*	In the last 24 h, 1–6 Likert scale
Hours of work in the last 24 h	*hoursOfWork_last24hs*	
Hours of free time in the last 24 h	*hoursFree_last24hs*	
Hours of sport in the last 24 h	*hoursSport_last24hs*	
Hours spent outdoors in the last 24 h	*hoursSpentOutdoors_last24hs*	
Hours spent directly interacting with electronic devices in the last 24 h	*hoursUsingScreens_last24hs*	
Hours spent in active social interaction in the last 24 h	*hoursActiveSocialInteraction_last24hs*	
Hours spent in passive social interaction in the last 24 h	*hoursPassiveSocialInteraction_last24hs*	
Positive and negative affect (PANAS scales)	*PANAS_[itemName]*	During the scanning. Individual questions are listed in the covariates data file
Sleep quality	*sleepQuality_lastNight*	1–6 Likert scale, with its extremes specified as 1: “Very badly” to 6: “Very well”
Can remember dreams from previous night	*rememberDreams_fromLastNight*	Binary variable (0/1)
Frequency of day dreams (mindwandering) in the last 24 h	*dayDreams_last24hs*	1–6 Likert scale
Time went to bed the previous night	*time_bed*	Measured with a FitBit activity tracker and completed manually in case of omission
Time spent in bed the previous night	*time_slept_min*	Measured with a FitBit activity tracker and completed manually in case of omission
Number of steps made in the last 24 h	*number_steps*	Measured with a FitBit activity tracker
Distance walked in the last 24 h	*walk_distance*	Measured with a FitBit activity tracker
Number of stories climbed in the last 24 h	*number_stories*	Measured with a FitBit activity tracker
Calories burned in the last 24 h	*calories_burned*	Measured with a FitBit activity tracker

These values are provided in the dataset as a text file with a JSON string. Unless otherwise noted in the table, only the extreme values of Likert scales were specified, from “None at all” to “Very much”

We measured MR Room temperature and humidity with a digital indoor weather station (WS9410, TechnoTrade, Wildau, Germany). We collected weather data retrospectively from the German Weather Service (Deutsche Wetterdienst, [27]). We used standard devices to measure blood pressure and body weight. We recorded responses to the PANAS scales [28] to measure positive and negative affect at the moment of scanning. We tracked physical activity and hours of sleep with a Fitbit One activity tracker (Fitbit, San Francisco, USA).

We collected saliva samples using SaliCaps collection devices (IBL-International, Hamburg, Germany), which are validated for sampling of steroid hormones. Immediately after collection, saliva samples were frozen and stored at −25 °C. Oestrogen and testosterone concentrations were determined with the Saliva ELISA kit (IBL-International, using IBL Saliva Immunoassay -17ß-Estradiol) and the IBL Saliva Testosterone Luminescence Immunoassay.

## Quality assurance

The technical image quality of the scanner was monitored by a quality assurance (QA) protocol defined after Friedman and Glover [29] including the Weisskoff noise coherence parameters [30]. Our QA protocol mainly focuses on scanner stability, signal-to-noise, drift, ghosting and other performance issues related to MR scanners. It was measured once per week, throughout the whole study period between July 2013 and February 2014. A BIRN agar phantom with 17-cm diameter was used.

Parameters that can be estimated in the Glover stability QA protocol include a signal image, temporal fluctuation noise image, signal-to-fluctuation-noise ratio (SFNR) image and a summary SFR value, a static spatial noise image, a signal to noise ration summary value, percent fluctuation and drift, a Fourier analysis of the residuals, and a Weisskoff analysis. Regular measurements of SNR, SFNR, percent fluctuations, and drift can provide critical feedback regarding scanner performance. The Fourier analysis can reveal periodic noise in time-series. Drifts in the RF amplifier gain settings and resonant frequency can also provide valuable feedback about the state of the scanner.

There were no major hardware or software changes made during the study period. There were no software upgrades as the scanner at our site is used solely for research purposes with numerous longitudinal studies, enabling a focus on continuity and therefore a neglect of software upgrades.

## Utility and discussion

This dataset will allow researchers to quantify intra-individual variability in different measures of brain structure and function, characterize its time course, and identify its potential sources. It constitutes a unique effort to sample MR data multiple times from the same individuals, covering the variability over time, individuals and measurement variables that Cattell identified [21, 22]. It includes brain data over 40–50 measurement occasions for more than one person, and an array of time-varying covariates.

While several software tools are already available for the pre-processing and statistical analysis of MR images, new tools are also being developed. This dataset can also be of use to test the stability and sensitivity of measures of brain structure and function obtained through newly developed tools.

We have carried on analyses of these data and that we plan to publish in four different manuscripts. One article focuses on the reliability of measures of functional connectivity in resting-state [31] and a second article presents an analysis method to study functional brain dynamics (also on resting state data) on an individual level [32]. Two additional articles (currently in preparation) will quantify and describe the within-subject variability in structural measures.

Perhaps the main limitation of the dataset is that, due to the challenges of data collection, it includes a rather small and homogenous group of participants, compared to other MR studies that are focused on inter-individual differences. However, analyses of this relatively homogenous dataset might provide compelling evidence and arguments to extend this approach to larger, more heterogeneous populations. We recommend collecting a similar dataset in other age groups to examine the tenability of the ergodicity assumptions data across the lifespan.

## Conclusion

This dataset, unique in the field of MRI, will allow researchers to address the important dimension of intra-individual variability in MR images. Because behavioural and physiological measures are included, multi-variate analyses can be performed to identify potential sources of variability. This dataset can therefore be used to inform MRI studies, in general, about potential confounds that should be taken into account when collecting and analysing MR data.
